# Archaea Signal Recognition Particle Shows the Way

**DOI:** 10.1155/2010/485051

**Published:** 2010-06-28

**Authors:** Christian Zwieb, Shakhawat Bhuiyan

**Affiliations:** ^1^Department of Molecular Biology, University of Texas Health Science Center at Tyler, 11937 US Highway 271, Tyler, TX 75708-3154, USA; ^2^Department of Biology, Division of Business and Sciences, Jarvis Christian College, P.O. Box 1470, Hawkins, TX 75765, USA

## Abstract

Archaea SRP is composed of an SRP RNA molecule and two bound proteins named SRP19 and SRP54. Regulated by the binding and hydrolysis of guanosine triphosphates, the RNA-bound SRP54 protein transiently associates not only with the hydrophobic signal sequence as it emerges from the ribosomal exit tunnel, but also interacts with the membrane-associated SRP receptor (FtsY). Comparative analyses of the archaea genomes and their SRP component sequences, combined with structural and biochemical data, support a prominent role of the SRP RNA in the assembly and function of the archaea SRP. The 5e motif, which in eukaryotes binds a 72 kilodalton protein, is preserved in most archaea SRP RNAs despite the lack of an archaea SRP72 homolog. The primary function of the 5e region may be to serve as a hinge, strategically positioned between the small and large SRP domain, allowing the elongated SRP to bind simultaneously to distant ribosomal sites. SRP19, required in eukaryotes for initiating SRP assembly, appears to play a subordinate role in the archaea SRP or may be defunct. The N-terminal A region and a novel C-terminal R region of the archaea SRP receptor (FtsY) are strikingly diverse or absent even among the members of a taxonomic subgroup.

## 1. Introduction

Protein sorting fundamentally maintains the identity and function of every cell with participation of the signal recognition particle (SRP). SRP components have been found in nearly all organisms [1]. Except in chloroplasts, SRP is a ribonucleoprotein [2]. The SRP RNA is typically composed of about 300 nucleotide residues and forms a complex with an extraordinarily conserved protein named SRP54 in archaea and eukarya or Ffh (fifty-four homolog) in the bacteria. A 19 kDa protein, SRP19, is present in archaea and eukarya, but absent in the bacteria. Polypeptides which are homologous to the eukaryal SRP9/14 and SRP68/72 heterodimers have not been found in the archaea genome sequences giving rise to an archaea SRP which is dominated by RNA [3, 4].

**Figure 1:**
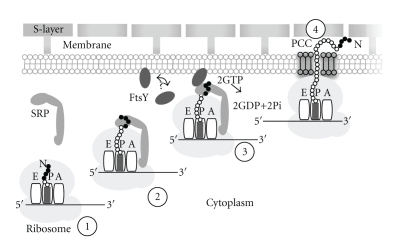
Hypothetical steps in the SRP-mediated targeting of archaea proteins. Step 1: A ribosome (gray, with A, P and E tRNA binding sites) in the cytoplasm translates a mRNA molecule (black, 5′ and 3′ ends are indicated) which encodes a N-terminal signal or membrane-anchor sequence (black dots). Step 2: As the signal emerges from the large ribosomal subunit, it is recognized by the elongated SRP and further translation may be halted. Step 3: The SRP-bound ribosome nascent chain complex (RNC) binds to free or membrane-associated FtsY (arrow). Step 4: After GTP hydrolysis, SRP has been released, translation resumes, and the targeted protein is threaded through the protein-conducting channel (PCC). The surface (S) layer, present in most archaea, is anchored to a glycerol-ether lipids-containing cell membrane.

SRP interacts with secretory signal or membrane-anchor sequences upon their emergence from the ribosomal exit tunnel. In vitro and in vivo experiments carried out in eukaryotic protein sorting systems have shown that the SRP delays or blocks the translation of the to-be-targeted polypeptides. Translation resumes when the SRP-bound ribosome nascent chain complex (RNC) binds to the membrane-associated FtsY (filamentous temperature sensitive Y) or, in eukaryotes, the alpha subunit of the SRP receptor (SR*α*). The interaction between SRP54 and the SR increases the affinity of the proteins for guanosine triphosphate, promotes the release of the signal from the SRP, and interjects the signal sequence of the nascent polypeptide into the protein-conducting channel (PCC) of the cell membrane. Translation and protein translocation or membrane insertion take place during ongoing translation (cotranslational translocation), and, upon hydrolysis of two GTP molecules, the SRP returns to its free cytosolic state ready to initiate another protein targeting cycle (Figure 1) [5–9].

Even though archaea membranes differ significantly from the cell membranes of eukaryotes and bacteria with regard to the use of glycerol-ether lipids and isoprenoid side chains [10, 11], no obvious adaptations for survival under extreme conditions are apparent in the SRP components. Like bacteria, archaea contain only one SRP receptor polypeptide, FtsY, a homolog of the eukaryal SR*α* subunit. The signal sequences of the archaea and bacteria are interchangeable [12, 13], and archaea signal peptidases which remove the signal sequence after protein translocation have been identified [14]. Archaea lack homologs of the bacterial and eukaryal translocation ATPases SecA and Kar2p/BiP. They possess, however, Sec61 (the PCC) and a Tat translocase system [15]. These SRP independent means of protein delivery have been discussed recently [16, 17] and will not be reviewed here. 

Solving the structures of numerous archaea SRP components and their complexes at high resolution (Table 1) has been crucial for understanding the intricacy of protein targeting in all organisms. Within this structural framework, the increasing number of newly identified archaea genome sequences provides an opportunity to review and discover not only archaea-specific SRP features, but also draw phylogenic distinctions which may pave the way for a better understanding of the function and evolution of every SRP.

## 2. Archaeal SRP RNA

**Figure 2:**
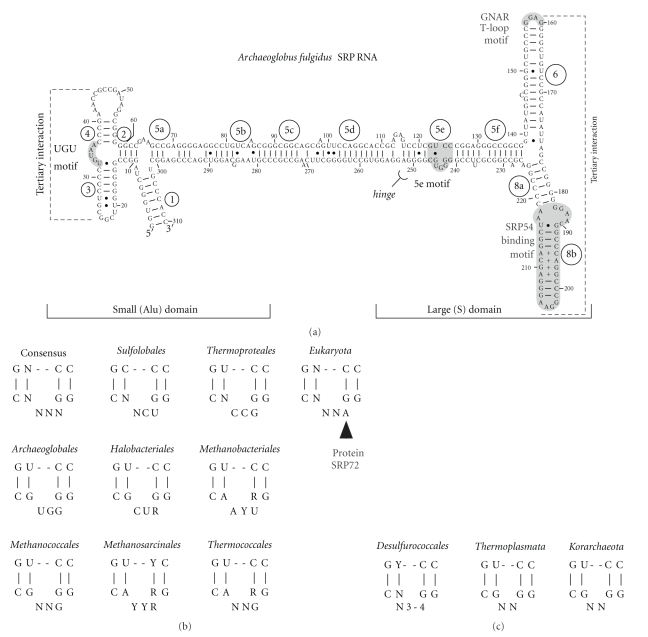
Archaea SRP RNA features. (a) Secondary structure of *Archaeoglobus fulgidus* SRP RNA. (b) Consensus-matching 5e motifs sorted by their taxonomic membership. (c) Mismatching 5e motifs. Helices are numbered from 1 to 8 and helical sections are labeled with letters a to f [33]. For example, helix 1 is composed of residues one to seven which are base paired with residues 303 to 310; helix 2 consists of residues at positions ten to 13 base paired with the residues at positions 59 to 62. The extended helix 5 contains six helical sections, 5a to 5f. Helix 7 is lacking in the SRP RNAs of the archaea. The 5′- and 3′-ends are shown, and residues are labeled in ten-residue increments. Base pairs were determined by comparative sequence analysis [32] and by considering high-resolution data (Table 1). The approximate extents of the large (S) and the small (Alu) domains are indicated. Shown in dark gray are the UGUNR motif (labeled UGU) in the small domain, the 5e motif within helical section 5e at the indicated hinge [34], the GNAR apical tetraloop of helix 6 and the SRP54 binding motif of helix 8 in the large domain. Dashed lines suggest tertiary interactions.

Unlike the bacterial and eukaryal SRP RNAs, their archaea counterparts vary little in shape and size (approximately 300 nucleotide residues). This may be due to relatively slow evolutionary rates as has been observed previously when the relative conservation of archaea protein sequences was investigated [31]. Archaea SRP RNA secondary structures possess extensively base paired regions which form a prominent central helix flanked by a small (or Alu) and a large (or S) domain (Figure 2(a)). Thus, they resemble the secondary structures of the mammalian SRP RNAs. Helices have been assigned numbers from one to eight, and helical section are designated with letters a to f [32, 33]. The SRP RNAs of most archaea as well as certain bacteria (e.g., Bacilli and Clostridia) pair their terminal regions to form a helix 1. Helix 7 has been found only in eukaryal SRP RNAs where it is most prominent in some fungi and protozoans [1].

Using the previously described sequence identification procedures and covariation rules (see Methods) we aligned 81 archaea SRP RNA sequences and arranged them according to NCBI's taxonomy [35]. The shared alignment pairing mask allows to deduce phylogenetically supported SRP RNA secondary structures for each of the aligned sequences. With a few exceptions, a sequence corresponds to a known species.

The apical loops of SRP RNA helices 3 and 4 form a tertiary interaction which is well supported by covarying compensatory base changes. The UGUNR sequence motif (N is A, C, G or U, R is a purine) located between these helices (labeled UGU in Figure 2(a)) is part of a structurally important U-turn. Both features promote the high degree of compactness of the small SRP domain. It remains to be determined how similar the structure of the protein-free small domain of the archaea SRP is to the solved crystal structure of the mammalian Alu domain in complex with the SRP9/14 protein heterodimer [36]. 

As previously noted and confirmed by mining of the larger collection of archaea SRP RNA sequences, deviations from the UGUNR motif occur in several groups [37]. Conspicuous erosions of the small domain take place in the SRP RNAs of several Desulfurococcales and in *Nitrosopumilus maritimus* SCM1. Base pairs which typically participate in the formation of helices 1 and 3 are absent in these sequences, while other residues perhaps form an extended helix 4. Due to the relatively small number of available sequences within these subgroups it is not yet possible to conclusively prove or disprove plausible base pairs.

Another hydrogen-bonded tertiary interaction engages two adenosines within the apical tetraloops of helices 6 and 8 (A159 and A205 in *Archaeoglobus fulgidus*, Figure 2(a)). This long-range interaction was first seen in the crystal structures of *Methanococcus jannaschii* SRP RNA from the large domain (Table 1). The adenosine clamp severely constraints the arrangement of helices 6 and 8. It is highly conserved and likely exists in all archaea and eukaryotic SRP RNAs. The participating adenosine of helix 6 is presented within a GNAR tetranucleotide loop (tetraloop) in most archaea SRP RNAs, but deviates (AAAG) from the consensus in the four SRP RNA sequences of the Thermoplasmatales. The interacting helix 8 has a GRRA loop with GGAA being the most frequently represented tetranucleotide. GGGA is found in the Thermoproteales and Thaumarchaeota (Nitrosopumilales), and GAGA in the Methanopyrales. These helix 8 tetraloop sequences are probably useful when attempting to identify and classify the archaea SRP RNAs (Table 2).

## 3. The 5e Motif: A Case for Molecular Exaptation

The 11-nucleotide 5e element is the most recently discovered SRP RNA motif and has been helpful in the prediction of SRP RNA genes [38]. The motif consists of four base pairs interrupted by a three-nucleotide loop. Two of the base pairs are symmetrically arranged G-C pairs. The comparison of 141 eukaryal and 28 archaea sequences shows that the first residue of the eukaryotic 5e loop is a conserved adenosine (A240 in human SRP RNA) in the eukarya (Figure 2(b)) [39]. In the archaea, the corresponding nucleotide can be any residue, and only two halobacterial sequences (*Haloferax volcanii*, GenBank Accession AF395888, and *Halomicrobium mukohataei*, GenBank Accession CP001688) possess an adenosine.

Systematic site-directed mutagenesis of the 5e region showed that human SRP RNA with a single A240G change was unable to form a complex with full-length human SRP72 [39]. The 5e RNA was found to bind a 56 amino acid-residue polypeptide of human SRP72 which contained the consensus sequence PDPXRWLPXXER (X is for any amino acid residue) [40]. Bioinformatic analyses identified two relatively poor consensus sequence matches in the genomes of archaea, one with a methyl coenzyme M reductase of an uncultured methanogenic archaeon (GenBank Accession ABI18429), the other with a hypothetical protein of *Pyrobaculum islandicum* DSM 4184 (GenBank Accession ABL88435). These relationships are likely coincidental and, until proven otherwise, are consistent with the notion that a functional equivalent of the eukaryotic SRP72 is lacking in the archaea.

The conserved adenosine in the 5e motif of the eukaryal, but not the archaeal SRP RNAs suggests that the 5e element was recruited in evolution to supply a new function to the protein-rich eukaryotic SRP thereby providing a striking example for molecular exaptation, defined as the utilization of a feature for a function which differs from what it was originally developed for [41, 42]. Because human SRP72 binds strongly to the *Haloferax volcanii* SRP RNA [40], the structures of the 5e region of archaea and eukaryotes are apparently very similar.

The 5e RNA fragment is remarkably resistant towards ribonucleolytic attack [39] indicating that it is compactly folded and may resemble the structure of an RNA kink-turn [43]. Although 5e conforms only loosely to the K-turn consensus secondary structure, 3D molecular modeling demonstrates compatible structures (Zwieb, unpublished). This suggest that 5e is part of the bend or hinge which allows the elongated SRP to adjust to the curvature of the ribosome and bind simultaneously to separate ribosomal sites [34]. Such an interpretation is supported by the finding that 5e is present in SRP RNAs with a standard set of helices in their small SRP domain [38]. Conversely, Figure 2(c) and the data shown in Table 2 suggest that SRP RNAs deviate from the 5e consensus when they lack the UGUNR motif or when the small SRP domain is eroded. These hinge-impaired archaea SRP RNAs may function in a mode which resembles the SRP-mediated protein targeting of the majority of bacteria which lack the small SRP domain.

## 4. Protein SRP19, Is It Required?

**Figure 3:**
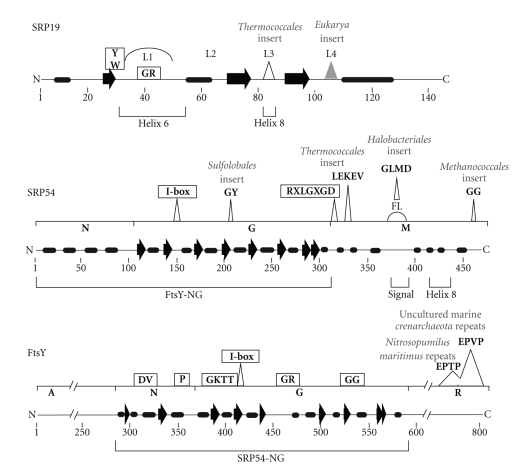
Features of SRP19 (top), SRP54 (center) and the FtsY SRP receptor (bottom). Indicated are the N- and C-termini. Helices are shown as cylinders, beta-sheets as arrows, and some loops are labeled with an arch and the letter L. Invariant or highly conserved residues are shown boxed, and several amino acid residue inserts which are characteristic for the indicated taxonomic groups are shown as bold letters. Numbering is according to the column positions of each protein alignment accessible as listed in Supplementary Materials 2 and at the SRP database at http://rnp.uthct.edu/rnp/SRPDB/SRPDB.html). Regions and sites which interact with other SRP components or the signal sequence are marked with brackets below each panel.

Although protein SRP19 was thought to be absent in certain archaea genomes [3], its genes (91 sequences) have now been identified in all archaea subgroups (Table 2). SRP19 coexist with SRP RNA helix 6 as part of the large SRP domain. Mainly due to the reduced size of its loop 4, the archaea SRP19 is generally somewhat shorter than its eukaryotic homolog (Figure 3, top, gray triangle). The NMR structure of *Archaeoglobus fulgidus* has been solved [23], and several crystal structures of the free and RNA-bound SRP19 have been determined (Table 1) revealing a single-domain compactly folded protein.

Certain conserved amino acid residues (Y/W and GR in loop 1; Figure 3, top) participate in the binding to the SRP RNA through induced fit mechanisms involving both the protein and the RNA. For example, loop 3 (Figure 3, top) of *Archaeoglobus fulgidus* SRP19 reorders and adopts a single conformation upon binding to RNA [23]. In the Thermococcales, loop 3 is enlarged and disordered and, upon binding, rearranges to assist in the proper folding of the SRP RNA [29]. This mechanism of mutual conformational adjustment has been observed in several other protein-RNA complexes [44].

In eukaryotic cells, SRP is assembled in the nucleolus and transported to the cytosol where it associates with SRP54 [45, 46]. Archaea SRPs contain only two proteins, SRP19 and SRP54, and assemble in the cytosol. The mammalian SRP19 is required to position SRP RNA helices 6 and 8 in a side-by-side fashion and expose the SRP54 binding site through a conformational collapse in helix 8. In contrast, archaea SRP RNA binds SRP54 even in the absence of SRP19 [47, 48]. RNase susceptibility measurements of wild-type and mutant* Archaeoglobus fulgidus* SRP RNAs show that the conserved adenosine of the GNAR tetraloop in helix 6, and not SRP19, is responsible for a compactly arranged large SRP domain [49]. Indeed, helices 6 and 8 are closely packed in the protein-free crystal structures of *Methanococcus jannaschii* and *Sulfolobus solfataricus* SRP RNAs [22, 27].

**Figure 4:**
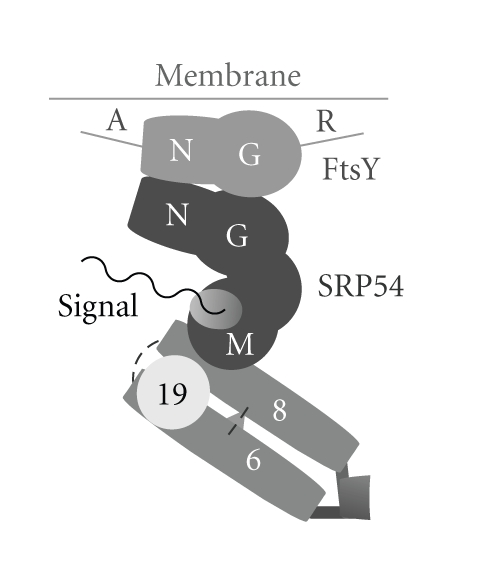
Interactions between the components of archaea SRP-mediated protein targeting. Schematic drawing of the coaxially-arranged SRP RNA helices 6 and 8 bound together by SRP19 and two tertiary interactions (dashed lines). The M-domain of the SRP54 protein (dark gray) binds to SRP RNA helix 8 as well as the signal sequence (black). The NG-domains of SRP54 and the FtsY SRP receptor are arranged quasisymmetrically and poised to separate upon the hydrolysis of two G-domain-bound GTP molecules. The N-terminal region labeled A (for acidic) and the C-terminal repeat region (R) of FtsY are variable or may be absent (see Table 2).

Figure 4 indicates that helix 6 and helix 8 interact with each other not only through their distal tetraloop adenosines but also via internal looped-out residues. However, the asymmetric internal loop of helix 8 engages in distinctly different ways. In the human SRP RNA, two adenosines protrude from the short strand of the asymmetric loop to form A-minor motifs with helix 6 [50]. In contrast, in the *Methanococcus jannaschii* RNA structures, two adenosines of helix 6 are bulged out and interact in the minor groove of helix 8 [25].

Deletion of the yeast SRP19 homolog Sec65 was shown to be lethal to the eukaryote *Yarrowia lipolytica* [51]. In the archaea, structural and biochemical data as well as the deviation from the GNAR tetraloop motif observed within the Thermoplasmatales (Table 2) suggest that SRP19 is not required for SRP assembly and dispensable for protein sorting and survival. In fact, deletion of SRP19 from the *Haloferax volcanii* genome had no effect on protein translocation or membrane insertion. Increased levels of membrane bacterioruberin were detected in the deletion mutant and significant amounts of SRP19 mRNA were observed in nonmutated cells [52] suggesting a relatively minor possibly regulatory function for SRP19. Although the protein might participate in a more substantive way when* Haloferax volcanii* is challenged to survive in external environments, the data demonstrate the diminished importance of the archaea SRP19 when compared to its significant role for the survival of eukaryotic cells.

## 5. SRP54

SRP54, or its bacterial homolog Ffh, is present in all organisms, including the chloroplast SRPs which lack an SRP RNA [2]. Deletion of the *Haloferax volcanii* SRP54 gene results in the loss of cell viability as proof of the central role of SRP54 in archaea protein targeting [52, 53]. Sequence and three-dimensional structure (Table 1) of the protein are highly conserved. These properties are readily explained by the numerous interactions which engage SRP54 in the binding not only to the SRP RNA, but also the signal sequence and the FtsY SRP receptor. The observed exceptionally high level of conservation likely reflects the need to carry out multiple binding reactions in a coordinated dynamically GTP-regulated way to ensure proper and efficient delivery of a wide variety of signal sequence-tagged proteins into the PCC.

The functions of SRP54 are brought about by three domains. The N-terminal (N) domain is composed of a bundle of four alpha helices, the GTPase (G) domain contains a unique insertion (I-box) which serves as a guanine nucleotide-exchange factors (GEFs) and stabilizes the nucleotide free protein [54, 55], and the methionine-rich (M) domain binds to the SRP RNA and the signal sequence (Figure 3, center). The predominantly alpha helical M domain contains an extended segment (the so-called fingerloop) which delineates or is folded into a groove which accepts signal sequences [20, 21, 56]. This wide and short hydrophobic groove was observed also in the crystal structure of the RNA-bound *Escherichia coli* Ffh [57]. The NMR structure of the *Archaeoglobus fulgidus* SRP54 M domain [24] is similar to these crystal structures and disfavors another proposed mode whereby the signal sequence binds within a long and narrow groove of SRP54M [28, 50]. The conformations of the fingerloop in solution suggest that it adaptively binds and stabilizes the signal sequences. Binding is weak [24] and likely reversible in order to permit signal sequence release upon the binding of SRP54 to the SRP receptor. The molecular details of the contacts made by a signal peptide with the *Sulfolobus solfataricus* SRP54 have been revealed recently and suggest that portions of the fingerloop may adopt an alpha helical conformation [21].

Adding to the intricacy of signal sequence recognition, the M domain and the NG region of SRP54 are joined together via a flexible linker. This region has the consensus sequence RXLGXGD and allows the RNA-bound SRP54 to undergo substantial structural rearrangements upon binding to a signal sequence [20, 22]. Consistent with this assertion, site-directed mutagenesis experiments of mammalian SRP [58] and a recent crosslinking study of the *Escherichia coli* SRP [59] demonstrate the involvement of the signal sequence not only with the M domain, but also the NG region. No evidence for the binding of NG to signal sequences has been provided in the archaea. However, the exceptional conservation of SRP54 throughout all domains of life suggests that archaea employ a similar if not identical signal recognition mechanism. The NG region can be in close proximity to SRP RNA helix 8 and, in archaea, appears to engage also helix 6 [25].

The alignment of 103 archaea SRP54 sequences reveals several group-specific amino acid residue insertions, for example a GY in the G domain of Sulfolobales which might modulate the GTPase activity. Into the M domain, Thermococcales insert the sequence LEKEV, Halobacteriales GLMD, and Methanococcales GG (Figure 3). These amino acid residues have the potential to contribute to the binding of the protein to the SRP RNA, to signal peptide recognition or other yet to be specified enhanced functions. Regardless of their potential significance, these short peptide sequences are useful for assigning SRP54 sequences to their proper taxonomic group.

## 6. FtsY: The SRP Receptor

The SRP receptor (SR) of the eukarya is composed of the peripheral membrane SR*α* and the integral membrane SR*β* proteins. Bacteria and archaea possess only FtsY, a homolog of SR*α* [60]. Sequence comparisons of FtsY with SRP54 suggest a gene duplication event [61] and support the classification into the three domains of life as well as the close rooting of archaea and eukarya [62]. Archaea FtsY shares its conserved NG region with NG of SRP54, including the I-box, but differs from SRP54 with respect to several short amino acid stretches as revealed by the alignment of 95 archaea FtsY sequences (Figure 3, Supplementary Material 1). The NG regions are symmetrically arranged in three dimensions to constitute the structural and functional core of signal sequence release and nascent polypeptide delivery into the cell membrane (Figure 4) by mutually catalyzing the hydrolysis of GTP [63–65].

As has been observed within the bacterial genomes [66, 67] several archaea FtsY sequences consist only of the NG domain and lack an N-terminal acidic (A) domain. Diversity with respect to the A domain is observed even within a single archaea subgroup (Table 2). Full-length *Haloferax volcanii* FtsY as well as polypeptides lacking the A domain were shown to bind to inverted membrane vesicles indicating that the A domain is dispensable for attaching FtsY to the membrane. Instead, the A domain may play a role in recruiting SRP to the haloarchaeal membrane [68, 69]. Assuming a pool of free FtsY in the cytosol [70, 71] (Figure 1) these findings are particularly relevant. On the other hand, fluorescence microscopy showed that almost all of the *Escherichia coli* FtsY associates in vivo with the inner membrane, and any soluble FtsY is unlikely to contribute to protein targeting [72]. Although archaea FtsY might interact with the membrane in similar manners as has been observed in bacteria and chloroplast [2, 73–76], the molecular details of the binding could be quite different given the differences in membrane lipid composition. FtsY might also interact directly with a cytosolically exposed portion of the PCC [77, 78]. In either case, one would expect functional synchronicity between GTP hydrolysis and delivery of protein into the PCC [79].

In the FtsY sequences of the uncultured marine Crenarchaeota we discovered a C-terminal proline-rich extension, named R for its motif repetitions (see Figures 3 and 4). Up to 12 EPVP repeats (accession numbers ABZ10052, ABZ08863, ABZ09152, ABZ09615) and five EPVV repeats (ABZ098531) were present in the R region. Similar multiple repeats with the sequence EPTP were seen also in the FtsY of the Thaumarchaeotum *Nitrosopumilus maritimus* SCM1. Details of the R-regions can be inspected in an updated archaeal FtsY alignment provided at the SRPDB [37]. As with much of our limited understanding of the role of FtsY in the archaea, it remains to be determined if these repeats are expressed and have a function in protein export.

## 7. Archaea SRP Function and Evolution

During the past years, several interesting puzzle pieces with respect to SRP-mediated protein translocation and membrane insertion in the archaea have been assembled. The SRPs of the Crenarchaeotum *Acidianus ambivalens* and the Euryarchaeota *Archaeoglobus fulgidus*, *Pyrococcus furiosus* and *Haloferax volcanii* have been reconstituted [47, 48, 80–82], and the ability of an archaea SRP54 to participate in signal sequence recognition has been demonstrated [81]. Nevertheless, the role of SRP within the archaeal cell is still poorly understood. Examples of both protein synthesis-linked (cotranslational) and posttranslational translocation have been provided [83–87], but to what degree these findings are representative remains to be investigated further [16]. 

The proposal that signal sequences might interact with the SRP RNA has fed the imagination that the primitive SRP was composed only of RNA [57, 88]. However, because of the proteinaceous nature of the signal, a scenario in which SRP RNA coemerged with evolutionary precursors of SRP54/Ffh/FtsY appears to be more plausible. Furthermore, the recent structure of the signal peptide-bound *Sulfolobus solfataricus* SRP54 (Ffh) shows that the signal peptide is too far removed from the SRP RNA to make direct contact [21].

If the small (Alu) SRP domain was a feature of the primitive SRP which subsequently was lost in evolutionary time; the majority of the bacteria is more difficult to discern. As another possibility archaea and certain bacteria may have been faced independently with the need to enlarge a small primitive SRP, maybe to slow down translation rates and provide more time for ensuring the delivery of proteins to the membrane as has been observed in eukarya [89].

## 8. Future Directions

With respect to the RNA-rich archaea SRP it would be desirable to better understand the structure and function of the protein-free small SRP domain. For example, what, in molecular detail, allows the small domain to fold back onto helix 5 in order to approximate the shape and dimensions of the eukaryal SRP [36]? What is the functional significance of the conserved 5e motif and its relationship to a flexible hinge or a bend in the elongated SRP? It will also be important to further elucidate the role of the archaea FtsY, its role in the cytosol as well as the molecular features which promote its association with archaea membranes. As in the past, the studies of the archaea SRP are expected to contribute in many ways to our grasp of SRP-mediated protein targeting in all organism.

## 9. Methods

Sets of representative sequences were used as input to Perl scripts written to identify sequence homologs in the NCBI databases [90]. RNA sequences were aligned semiautomatically with SARSE [91]; protein sequences were aligned using MUSCLE [92] followed by manual adjustments in Jalview [93]. The alignments are available through the links listed in Supplementary Material 2. In addition, the SRP database provides tables of alphabetically and phylogenetically sorted sequences at http://rnp.uthct.edu/rnp/SRPDB/SRPDB.html.

## Supplementary Material

Supplementary Material 1: Table of the features of archaea SRP proteins SRP19, SRP54 and FtsY receptors extracted from the protein alignments.
Supplementary Material 2: List of links to the SRP database (SRPDB) as well as the alignment and table files for the archaea SRP_RNA, SRP19, SRP54 and FtsY receptor.

## Figures and Tables

**Table 1:** High-resolution structures of archaeal SRP components. Indicated are the archaea subdomains (Crenarchaeota or Euryarchaeota), species names, components, and methods (X-Ray diffraction of NMR) used for structure determination. The pdb IDs allow easy retrieval of the coordinates [18]. The protein-conducting channel is abbreviated as PCC. Additional nonarchaea SRP high-resolution structures are listed at http://rnp.uthct.edu/rnp/SRPDB/srpstructures.html.

Subdomains	Species	Components	Methods	pdb	References
Crenarchaeota	*Acidianus ambivalens*	SRP54NG	X-Ray	1J8M,1J8Y	[19]
	*Sulfolobus solfactaricus*	SRP54 with helix 8	X-Ray	1QZW	[20]
		SRP54 dimer	X-Ray	1QZX	[20]
		SRP54 with signal peptide	X-Ray	3KL4	[21]
		SRP19 with helix 6 and helix 8	X-Ray	3KTV,3KTW	[22]
Euryarchaeota	*Archaeoglobus fulgidus*	SRP19	NMR	1KVN,1KVV	[23]
		SRP54M	NMR	2JQE	[24]
	*Methanococcus jannaschii*	SRP19 with SRP54 with RNA	X-Ray	2V3C	[25]
		SRP19 with helix 6 and helix 8	X-Ray	1LNG	[26]
		Helix 6 and helix 8	X-Ray	1Z43	[27]
		PCC	X-Ray	1RHZ,1RH5	[28]
	*Pyrococcus furiosus*	SRP19	X-Ray	3DLU,3DLV,3DM5	[29]
		SRP54	X-Ray	3DLU,3DLV,3DM5	[29]
		FtsY	X-Ray	3E70,3DM9,3DMD	[30]

**Table 2:** Taxonomic distribution of archaea SRP features. Indicated are the archaea subdomains, the number of species identified in each group, and a representative species. Features are the UGUNR motif (N is any nucleotide and R is a purine residue), helices (typically 1 to 4) in the small SRP domain (SD), the GNAR tetranucleotide loop (tetraloop) of helix 6, the GGAA tetraloop of helix 8, proteins SRP19 and SRP54 (SRP19/54), and the acidic (A) domain of the FtsY receptor (FtsY-A). “+” shows presence, “−” absence, and “±” indicates that this feature is present only in a subset of the group members. Sequences deviating from the top-listed motif (e.g., the GGGA in the Thermoproteales) are given. The structural alignments of the 81 identified archaea SRP RNAs and the protein alignments of SRP19, SRP54 and FtsY are provided online as listed in Supplementary Materials 2 available online at doi:10.1155/2010/485051and are available at http://rnp.uthct.edu/rnp/SRPDB/srprna.html. Features of the SRP RNA in all three domains of life have been recently described in detail in [1].

Subdomains	Groups (n)	Prototypical Species	UGUNR	SD helices	GNAR	GGAA	SRP19/54	FtsY-A
Crenarchaeota	Desulfurococcales (5)	*Pyrodictium occultum*	−	some absent	+	+	+	−
	Sulfolobales (12)	*Sulfolobus solfataricus*	±	+	+	+	+	+, varies
	Thermoproteales (4)	*Thermoproteus neutrophilus V24Sta*	−	+	+	GGGA	+	−
Euryarchaeota	Archaeoglobales (2)	*Archaeoglobus fulgidus*	+	+	+	+	+	±
	Halobacteriales (10)	*Halobacterium species NRC-1*	−	+	+	+	+	±, some long
	Methanobacteriales (3)	*Methanobacterium thermoautotrophicum*	+	+	+	+	+	+, some long
	Methanococcales (12)	*Methanocaldococcus jannaschii DSM 2661*	+	+	varies	+	+	±
	Methanomicrobiales (1)	*Methanoculleus marisnigri JR1*	UAUAA	+	+	+	+	±
	Methanosarcinales (5)	*Methanosarcina acetivorans*	+	+	+	+	+	±
	Methanopyrales (1)	*Methanopyrus kandleri AV19*	+	+	+	GAGA	+	−
	Thermococcales (14)	*Pyrococcus horikoshii OT3*	+	+	+	+	+	±, some long
	Thermoplasmatales (3)	*Thermoplasma acidophilum*	−	+	AAAG	+	+	−
Korarchaeota	Candidatus Korarchaeum (1)	*Candidatus Korarchaeum cryptofilum OPF8*	−	+	+	+	+	−
Thaumarchaeota	Nitrosopumilales (1)	*Nitrosopumilus maritimus SCM1*	−	some absent	+	GGGA	+	−
