# Supplementary material for: Archaea Signal Recognition Particle Shows the Way
Source: Archaea. 2010 Jun 28;2010:485051. doi: 10.1155/2010/485051 (PMC2905702; doi:10.1155/2010/485051)
Supplement: Supplementary file 1 — Supplementary Material 1: Table of the features of archaea SRP proteins SRP19, SRP54 and FtsY receptors extracted from the protein alignments. Supplementary Material 2: List of links to the SRP database (SRPDB) as well as the alignment and table files for the archaea SRP_RNA, SRP19, SRP54 and FtsY receptor. [file 485051.f1.pdf]

Supplementary Material 1

SRP protein	Features	Positions
SRP19	Y/W	29
	GR	42-43
	Loop 1	29-55
	Loop 2	67-69
	Thermococcales insert L3	82-86
SRP54	I-box	148-147
	Sulfolobales insert GY	208-210
	RXLGXGD	308-314
	Thermococcales insert LEKEV	325-329
	Halobacteriales insert GLMD	381-384
	Fingerloop	375-395
	Methanococcales insert GG	466-467
FtsY	DV	318-319
	P	355
	GKTT	397-401
	I-box	423-432
	GR	477-478
	GG	540-541
	Nitrosopumilus maritimus repeats EPTP	592-642
	Uncultured marine crenarchaeota repeats EPVP	645-684

Table. Features extracted from the alignment of archaea SRP proteins. The same features are shown in Figure 3. The column positions in the respective alignments of SRP19, SRP54 and FtsY are given in the right column.
