# Supplementary material for: Archaea Signal Recognition Particle Shows the Way
Source: Archaea. 2010 Jun 28;2010:485051. doi: 10.1155/2010/485051 (PMC2905702; doi:10.1155/2010/485051)
Supplement: Supplementary file 2 [file 485051.f2.pdf]

Supplementary Material 2. SRP database links

SRP database (SRPDB):

http://rnp.uthct.edu/rnp/SRPDB/SRPDB.html

Archaea SRP RNA lists and alignment file:

http://rnp.uthct.edu/rnp/SRPDB/rna/srprnaphylolist_arch.html

http://rnp.uthct.edu/rnp/SRPDB/rna/srprnaalphalist_arch.html

http://rnp.uthct.edu/rnp/SRPDB/rna/alignment/fasta/SRPRNA-archaea.fasta

Archaea protein SRP19 lists and alignment file:

http://rnp.uthct.edu/rnp/SRPDB/protein/srp19/srp19phylolist-arch.html

http://rnp.uthct.edu/rnp/SRPDB/protein/srp19/srp19alphalist-arch.html

http://rnp.uthct.edu/rnp/SRPDB/protein/srp19/alignment/fasta/srp19-arch_ali.fasta

Archaea protein SRP54 lists and alignment file:

http://rnp.uthct.edu/rnp/SRPDB/protein/srp54/srp54phylolist-arch.html

http://rnp.uthct.edu/rnp/SRPDB/protein/srp54/srp54alphalist-arch.html

http://rnp.uthct.edu/rnp/SRPDB/protein/srp54/alignment/fasta/SRP54-archaea.fasta

Archaea protein FtsY (SR-alpha) lists and alignment file:

http://rnp.uthct.edu/rnp/SRPDB/protein/sralpha/ftsyphylolist-arch.html

http://rnp.uthct.edu/rnp/SRPDB/protein/sralpha/ftsyalphalist-arch.html

http://rnp.uthct.edu/rnp/SRPDB/protein/sralpha/alignment/fasta/SRPFtsY-archaea.fasta
